# Total cannabidiol (CBD) concentrations and yields from traditional extraction methods: Percolation vs. maceration

**DOI:** 10.3389/fphar.2022.886993

**Published:** 2022-10-24

**Authors:** Jason Wilson, Travis Simpson, Kevin Spelman

**Affiliations:** ^1^ Health, Education & Research, Driggs, ID, United States; ^2^ Natural Learning Laboratories, Medford, OR, United States; ^3^ Massachusetts College of Pharmacy and Health Sciences, Boston, MA, United States

**Keywords:** Cannabis, extraction, cannabidiol, percolation, maceration, cannabinoids, medicinal plants, traditional medicine

## Abstract

Medicinal plants have been valued for many generations due to their biosynthetic advantages generating pharmacologically active molecules. This is especially the case when it comes to cannabinoids from Cannabis. In these experiments we mimicked typical herbal home extractions and measured the yield of total decarboxylated CBD (“total CBD”) from percolations and macerations done at the common duration of 2 weeks in duplicate independent extractions. Analysis was performed by GC-FID on triplicate samples from each extraction. Results demonstrated a significant extraction superiority of percolation over maceration. Percolation extracted 80.1% of the total CBD in the hemp biomass as compared to the 2-week time point at 63.5% recovery. Our results demonstrate a significant increase in total CBD yield from percolation, as compared to maceration. Highest solvent recovery was also through percolation, but overall solvent recovery was fairly consistent with the maceration method, after pressing. Under these conditions of extracting lipophilic cannabidiol in 95% ethanol, these data demonstrate that percolation is significantly superior to maceration in total CBD yield. These observations will likely apply to the extraction of lipophilic constituents from other herbs and botanical medicines.

## Introduction

Herbal medicines have been commonplace in Asia for many generations but have come back into fashion in Europe and North America with a 109.3% growth in sales in the US since 2000 (New Hope Network, 2019). Due to its pharmacological activity, Cannabis has become one of the botanicals in the public spotlight due to the change in regulatory structures in a growing number of countries, as well as the trail of politics following closely on its roots. Consumption of cannabidiol (CBD) chemotypes of *Cannabis sativa* L. [Cannabaceae]*,* known as hemp by consumers, has grown exponentially and now are at or above 34% of the population as of a 2022 survey (ICSID, 2022).

Hemp extracts containing CBD are used most commonly for chronic pain, arthritis, joint pain, and anxiety (Corroon and Phillips, 2018). This current use mimics the traditional use of hemp for pain and anxiety (Balant et al., 2021; Russo, 2007). The method of administration most commonly used are liquids administered as sprays, drops, and tinctures (Corroon and Phillips, 2018) and this reflects the broader choice of consumers for non-Cannabis herbal medicines as well. Extractions using ethanol, are common to Cannabis products and throughout the medicinal plant field in general. These types of extractions, called tinctures, are increasingly being made in home kitchens due to the relaxation of laws that previously prohibited growing Cannabis in residential gardens.

Investigations of traditional preparations of medicinal plants, such as hemp, offers insight into the phytochemical diversity and the yield of actives. Two dominant techniques are used throughout the medicinal plant community to make tinctures (WHO, 2017). The most common is maceration which mixes the properly comminuted herbal materials with the menstruum (most commonly a hydroethanolic solvent with varying ratios of water and ethanol) and allowing the mixture to stand at a certain temperature, usually room temperature, with agitation for a defined period of time. Preparation of macerations is documented by Paracelsus in the 1500s, who used extended maceration times in low percentage of ethanol in hydroethanolic solutions (Raubenheimer, 1910). The lesser used technique, but notably quicker, is percolation. This procedure provides a continuous flow of the menstruum through the herbal material in a percolation cone (WHO, 2017).

The manner in which natural medicines are prepared influences the final composition of the herbal remedy (Politi et al., 2005b; Politi et al., 2005a). This in turn provides insights into what consumers are ingesting, and of the potential pharmacological activity, as well as synergic pharmacological effects that may result from a complex extract.

There has been a long standing debate on the superiority of extraction methods for medicinal plants (Raubenheimer, 1910). Unfortunately, the scientific focus on the traditional methods of extraction have faded from pharmaceutical sciences due to the focus on new-to-nature molecules. There are very few pharmacognosy university study programs remaining throughout the world. This movement away from natural products for drug discovery is correlated with the overall reduction in new leads in the drug development pipeline and a substantial decline in new drug approval (Li and Vederas, 2009). Regardless, traditional preparations continue to be utilized on every corner of the planet by shamans, herbalists, naturopaths, modern phytotherapists and integrative physicians, yet with little substantiation of optimal extraction conditions.

In the following experiments we used the long-standing traditional method documented in the Eclectic dispensatory which suggests the use of “rectified” ethanol (95% EtOH) (Felter and LLoyd, 1898). Moreover, due to the highly lipophilic nature (log *P* > 6) 95% EtOH is commonly used in *Cannabis* production facilities around the globe to pull these lipophiles out of flower. To this end, we replicated different home extraction techniques that are traditional, as well as low tech, and that have been used by many generations of herbalists—using *Cannabis sativa* L. [Cannabaceae] as our botanical, and the phytocannabinoid cannabidiol (CBD) as our target active compound. We investigated the traditional extraction technique of maceration and percolation and compared this for total CBD yield. To our knowledge this is the first comparison of the extraction of actives by percolation and maceration of a medicinal plant and/or *C. sativa* L.

## Results

### Cannabis material characterization

The lot of decarboxylated Cannabis material was characterized through three repeated sample preparations for the purposes of calculating theoretical analyte recovery efficiencies. The Total CBD concentration mean, median, standard deviation, and percent relative standard deviation were calculated to understand the inherent heterogeneity of the material as well as the average CBD concentration to be used for calculating analyte recovery throughout the study (Table 1). The percent CBD of the biomass was calculated as 8.84% means in the biomass with a % RSD of 6.19.

**TABLE 1:** Milled Cannabis flower potency characterization.

Characteristic	Result in %
Rep 1 Means	9.36
Rep 2 Means	8.27
Rep 3 Means	8.90
Mean	8.84
Median	8.90
STD Deviation	0.55
% RSD	6.19%
*p* value	<0.002

Three samples were collected, and triplicate analyses were performed on each biomass sample for a total of 9 analysis to establish baseline biomass CBD concentration. Standard deviation was calculated for the 9 analyses. Comparison of means was conducted using a two tailed *t*-test for paired data when differences were observed, values were considered significantly different if *p* < 0.05. Differences were not significant.

### Yields of Cannabidiol

The percolation treatment achieved the highest recovery of CBD, with an average CBD recovery of 80.10, as compared to the 2-week maceration at 63.52% (Table 3). This demonstrates a high extraction efficiency for an ancient technique without any mechanical or thermal assistance: Approximately 20% of the theoretical total CBD present in the starting Cannabis biomass was left behind after the percolation treatment (Table 2). The 2-week treatment (63.52% average CBD recovery Table 3), resulted in 36.48% of the theoretical total CBD remaining in the biomass after maceration.

**TABLE 2:** CBD recovery by percolation.

Treatment	Total input Mass (g)	Total CBD mg input	Drain CBD (mg)	Total liquor CBD (mg)
Perc—1	53.8	4,757.974	3,772	3,772
Perc—2	53.8	4,757.974	3,850.3886	3,850.3886
Perc-Means	53.8	4,757.974	3,811.1943	3,811.1943

The CBD total milligrams were based on duplicate independent extractions with 3 samples for each extraction, analyses were performed in triplicate on each sample to calculate final CBD concentration.

**TABLE 3:** CBD recovery efficiency (% recovery) per extraction treatment.

Treatment	Total input Mass (g)	Input CBD mg/g	Total CBD mg input	Total liquor CBD recovery (%)	Drain CBD recovery (%)	Press CBD recovery (%)
2°weeks—1	100.073	88.438	8,850.274	61.922	53.573	8.348
2°weeks—2	100.067	88.438	8,849.743	65.108	57.085	8.023
Perc—1	53.8	47.580	4,757.974	79.277	79.277	0.000
Perc—2	53.8	47.580	4,757.974	80.925	80.925	0.000

The total percent recovery of CBD (drain + expressed liquid after pressing) and CBD from the drain (liquor collected without pressing) were significantly superior to the maceration by a means of 16.59% (*p* = 4.419 × 10^−9^). Pressing the marc (insoluble biomass) did not result in collection of any expressed liquid. CBD concentration based on duplicate independent extractions with three samples for each extraction, analyses were performed in triplicate on each sample to calculate CBD concentration. The means of the percolation analysis were then compared to the means of the maceration analysis.

Percolation was also the most precise method used, in that it delivered the most consistent results between treatment replicates, with only a mean 2.95% RSD between replicates. The 2-week treatment resulted in a mean 5% RSD.

### Pressing and Solvent Recovery

For menstruum recovery, the percolation treatment provided a modest advantage, with an average menstruum recovery of 75.19% as compared to an average of 70.26% of the pressed tincture. If the percolation menstruum recovery was compared to just the drained liquor without the expressed menstruum from the marc, the mean recovery of the liquor was 61.788% (Table 4), an improvement of 13.398%.

**TABLE 4:** Amount of ethanol recovered per extraction treatment.

Treatment	Starting solvent Mass (g)	Drain liquor Mass (g)	% Of original solvent	Press liquor Mass (g)	Total liquor Mass (g)	Liquor recovery %
2°weeks—1	500.120	297.490	59.483	41.660	339.150	67.814
2°weeks—2	500.190	320.580	64.092	43.040	363.620	72.696
Perc—1	269.000	205.000	76.208	0.000	205.000	76.208
Perc—2	269.000	199.502	74.164	0.000	199.502	74.164

The total liquor (menstruum) recovered was also significantly greater than in the maceration in the drain and percent of total liquor recovered. Duplicate independent extractions with 1 volume of liquor collected for each extraction.

## Discussion

These investigations include several limitations and opportunities for refinement. Resources were limited during the course of this project, which led to limits on the number of replicates for the extraction treatments to two replicates, when it would have been preferable to perform at least three replicates for adequate precision and accuracy. The amount of biomass used in percolation treatments also had to be limited to approximately 50 g due to the size of the percolation cone that was available, to mimic home extractions. Future follow up studies should match marc mass in the percolation and maceration experiments.

Further limitations of these investigations include the potential heterogeneity in CBD concentration of the biomass, potentially resulting in variable CBD concentrations in the starting biomass. Consistent distribution of biomass CBD content and particle size was managed by mixing the hemp biomass repeatedly and sifting the biomass through a #5 sieve for an effective medium approximation of CBD. Our analysis shows an RSD of [6.19%] for CBD concentration after triplicate samples of the hemp biomass and triplicate analysis of each sample.

In these experiments we compared the solvent recovery, yield of CBD, additional input of pressing marc in two disparate techniques of extractions; maceration and percolation. We also mimicked home extractions that a person not versed in laboratory extractions of medicinal plants could perform in a typical home kitchen. All items used; screen, oven, glassware and press, are either easily afforded in Western countries or are readily available in a typical household. In addition, the particle size of the flower, which had been baked at 250°F for decarboxylation, was consistent throughout both macerations and percolations.

These data show that in the maceration method, pressing the marc significantly increases the content of CBD in the menstruum. Our results show that an additional 8.19% (∼458 mg) of CBD was added to the menstruum from the pressing procedure. The step of pressing the marc to express the retained menstruum is often problematic for people making tinctures at home. We showed with an inexpensive and readily avialable wine press, that was not designed for pressing the biomass of tinctures, that there is a significant value in pressing the marc for an increase yield in active compound(s).

As opposed to menstruum recovery in the 2-week maceration, the total menstruum recovery in the percolation was improved by 4.931% as compared to the fully pressed macerated tincture and 13.398% of the unpressed macerated tincture. However, what most production facilities of medicinal plant tinctures employing maceration technique use is a press supported by a hydraulics. The above figures on menstruum recovery come from an inexpensive wine press. Thus, it is conceivable that a hydraulic press, generally not available in the herbalist’s kitchen would yield a higher menstruum recovery.

Interestingly, we attempted to press the marc from the percolation cone and did not recover any viable amount of menstruum (Table 4). Menstruum recovery for percolation was more efficient over maceration, even when pressing the macerated marc. These investigations show that percolation as an extraction technique, has a significantly increased extraction efficiency of CBD and menstruum from Cannabis tinctures as compared to a maceration.

Although the efficiency of percolation vs. maceration has been long debated, percolation is another extraction technique, traced back to ancient Greece, that has long been used for botanical medicines (Raubenheimer, 1910). Percolations are preferred by some medicinal plant extraction facilities because they are more time and space efficient. Instead of macerating solutions sitting for extended time periods in large drums, as traditionally done in the production of medicinal plant liquid extracts, percolations are finished in hours, rather than the traditional maceration time of 2–4 weeks. After two independent extractions the percolations extraction efficiency of CBD was 80.10% on average while the 2 week maceration extraction efficiency was 63.52% on average. This investigation provides data to supplement a long-held debate on the efficiency of percolations over macerations.

Percolation extraction requires a percolator, a cone shaped vessel narrowing at one end and open at both ends (Majeodunmi, 2015). Pharmaceutical grade percolation cones are not widely available for the home enthusiast. However, certain wine shaped glass bottles or sparkling water bottles can be adapted. In the attempts to keep this technique replicable to a consumer in a home kitchen we used a Perrier bottle, which is commonly used by herbalists, with the bottom of the bottle carefully scored and then removed. The plant material is scantly pre-moistened with the liquor, sits for 24 h and is then packed in a percolation chamber with the proper packing pressure. The liquor is then run through the packed herb in the percolator which has a stop cock at the end to control rate of flow, which depending on the herb, is from one to six drops a second (Raaman, 2006). A screw on cap on a Perrier bottle is often substituted in home extractions instead of a stop cock.

Another advantage of percolation may be preservation of phytonutrients. Select phytochemicals degrade more rapidly in a liquid medium than in a solid medium (Fairbairn et al., 1976; Rogers et al., 1998; Livesey et al., 1999; Perry et al., 2000; Bilia et al., 2002; Yang, 2008; Peschel, 2016). Phytocompounds in a macerating hydroethanolic solution, or sitting in tincture form on a shelf waiting for a consumer purchase, degrade on varying timescales. For example, caffeic acid derivatives, important herb constituents due to immunological influences (Kilani-Jaziri et al., 2017), have shown a remarkable ability to degrade in just a few months in hydroethanolic solutions (Livesey et al., 1999). Degradation in hydroethanolic solutions has also been shown for cannabinoids (Fairbairn et al., 1976; Peschel, 2016) and the lipophilic CB_2_ ligands known as the alkylamides (Livesey et al., 1999; Perry et al., 2000).

These results are particularly pertinent to other medicinal plant extractions in that the identical techniques used for Cannabis in these experiments are used for a wide variety of medicinal plants. Thus, these results may be pertinent to other lipophilic compounds that are extracted from medicinal plants. For example, in the case of *Echinacea* spp., one of the groups of active constituents are the alkylamides. Specific alkylamides bind with similar affinities to CB_2_ as the endocannabinoids (50–60 nM) (Matovic et al., 2007; Raduner et al., 2006) and have also shown PPARγ activity (Spelman, 2009). Previous research demonstrates that there is no difference in alkylamide concentrations in *Echinacea purpurea* roots extracted for 24 h *versus* 2 weeks in 70% ethanol (Spelman et al., 2009), however these results did not compare a percolated extraction.

The log P, a measure of lipophilicity, is less than ideal for optimal pharmacokinetics for both CBD and the alkylamides previously mentioned. Thus, these examples may be relevant to other lipophilic compounds in medicinal plants. Moreover, the topological polar surface area (PSA) for both compounds is also less than ideal for optimal pharmacokinetics suggesting that at the least, these two lipophiles may act similarly in crude extractions.

Finally, it should be noted that these extractions were carried out with CBD, not CBDA. Given that the PSA differs considerably between CBD (PSA = 40.5 Å^2^) and CBDA (PSA = 77.8 Å^2^) further extraction efficiency of CBDA in ethanol is expected due to the higher PSA. Under aqueous extraction conditions, superior extraction of tetrahydrocannabinolic acid (THCA) over tetrahydrocannabinol (THC) has previously been shown (Hazekamp, 2007). Thus, extraction from dried hemp that is not decarboxylated will likely be advantageous for extraction in ethanol.

To summarize, using common tools available in a home kitchen, an extraction performed by percolation has a statistically significant higher extraction yield on average of 16.59% CBD than a 2-week extraction (*p* = 4.419 × 10^−9^). In addition, the percolation method of extraction exhibited a superior performance of precision between replicates and menstruum recovery by 4.93% as compared to the combined liquor from the drain and pressed marc.

## Methods and materials

### Cannabidiol dominant Cannabis biomass

CBD dominant chemotype inflorescences were sourced from a local hemp cultivator (Rogue Farm, Central Point, OR) licensed by the Oregon Department of Agriculture (License ID: AG-R1043473IHH) as a hemp producer. *Cannabis sativa* L. [Cannabaceae] inflorescences were taxonomically identified by the Quality Control department. Inflorescences were trimmed, dried, and cured prior to receipt. To establish baseline CBD content triplicate samples of the biomass were taken. The three samples were analyzed in triplicate for a total of nine samples.

### Cannabis preparation methods

The inflorescences were decarboxylated at 250°F/121°C for approximately 2 h using a convection toaster oven. After decarboxylation, the material was milled (Shingle Manufacturing, High Tech Shredder 110 cup) for 15 s. The milled material was then sieved through a 4,000 μm, #5 mesh to remove larger particles to achieve a homogeneous batch of milled biomass. The material was then stored in an airtight stainless-steel container at room temperature (23°C ± 2°C).

### Extraction methods

Extractions were performed at room temperature (23°C) in duplicate and were controlled for all variables. All measurements performed throughout the extractions were verified by a second technician. Particle size was standardized as described above.

A menstruum ratio of 1:5, consisting of 100 g of milled Cannabis to 500 g of 95% organic ethanol (Organic Alcohol Company, Ashland, OR) was utilized for the maceration treatments. After 2 weeks the mixture was poured over a cheese cloth, into a 32 oz. wide mouth Ball canning jar. Once this primary menstruum was collected, the collected biomass in the cheese cloth was then pressed (EJWOX 0.53 Gallon Stainless Steel Press, Santa Ana, CA) for 5 minutes in order to collect a secondary “pressed” menstruum. The two menstruums were kept separate. Each maceration treatment was performed in duplicate.

For the percolation treatments, approximately 53.8 g of biomass saturated in 46.2 g of 95% organic ethanol (100 g total of same EtOH lot as above) were utilized from a batch of 300 g of biomass moistened with 258 g of ethanol (558 g total). The biomass was slightly moistened with the 95% ethanol per typical percolation protocol (Martin and Cook, 1961; Handa, 2008). While various authorities differ in the duration of time to moisten the biomass (Wood et al., 1907; Committee On The National Formulary VIII, 1946; Martin and Cook, 1961; Handa, 2008), a common tradition in the herbal community is 24 h (Moore, 1992). The following day (24 h later), the biomass was packed into the percolation cone and 269 g of ethanol was poured over the moistened biomass to reach a total of 222.8 g of ethanol used per percolation treatment. This ensured the same menstruum ratio of 1:5 was used, with a total of 222.8 g of menstruum to 53.8 g of hemp. Each percolation treatment took approximately 1 h to complete. Each percolation treatment was performed in duplicate. The rate of drip from the percolation cone was monitored to maintain one to two drop/second (Raaman, 2006). Once extracts were collected, they were labeled and stored in a laboratory freezer at approximately −36°C ± 2°C until they were accessed for analytical sampling.

### Analytical methods and quantification

The method utilized is a modified American Herbal Pharmacopoeia method (American Herbal Pharmacopoeia, 2013) and was validated according to ISO 17025 and TNI requirements for analytical method validation.

To establish CBD content in the extractions duplicate independent extractions were performed for each extraction. Three samples were taken for each independent extraction. In turn, triplicate analysis were performed on each sample.

Analytical samples were prepared by dissolving approximately 1 g of each sample in 20 ml of HPLC grade (>99% purity) methanol (Concord Technologies, Tianjin, China), agitating for no less than 10 min, and diluting 1/10th in methanol before transferring to 2 ml screw top vials.

**FIGURE 1:**
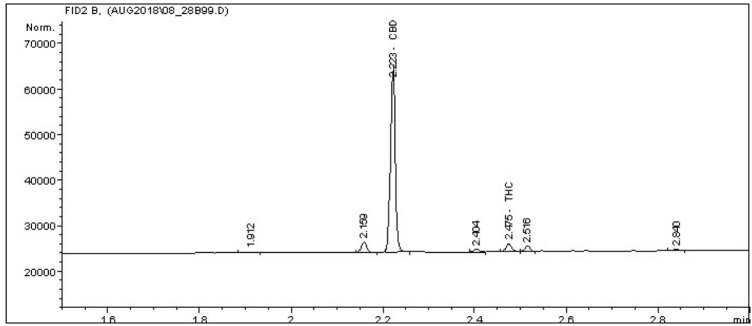
Calibration curves.

**FIGURE 2:**
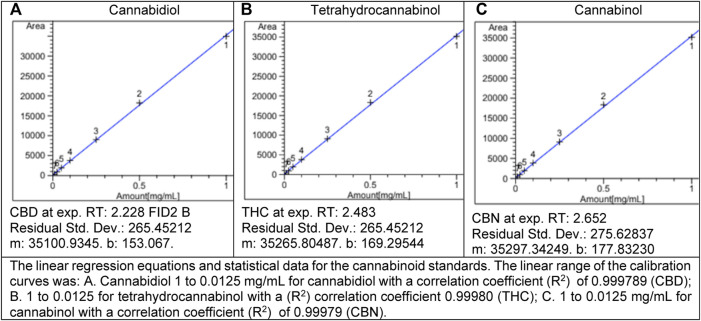
Sample chromatogram. A characteristic chromatogram obtained by gas chromatography–flame ionization detection of a *C. sativa* inflorescence extract, chemotype CBD. The chromatogram shows a peak chromatogram of CBD at 2.223 min with a minor peak for tetrahydrocannabinol (THC) at 2.475.

Analysis of total CBD was performed *via* gas chromatography with flame ionization detection (GC-FID) HP/Agilent 5,890 Series II with Agilent 7,673 Autosampler equipped with a 15 m long, 0.25 mmID, 0.25 μm capillary column (Restek Rxi-35Sil MS). Data acquisition and review was performed on Agilent Chemstation ver. A.10.02 software (Figure 1). A three-part THC, CBD, and CBN reference standard (Restek cat. #: 34,014) was used to establish seven calibration points at 1, 0.5, 0.25, 0.1, 0.05, 0.025, and 0.0125 mg/ml for the cannabinoids (Figure 2). This calibration was then confirmed using a second source standard which also contained THC, CBD, and CBN (Cerilliant cat. #: T-108-0.5ML). A continuing calibration verification sample containing 1 mg/ml of each target analyte was used to monitor calibration status before and after each analytical batch.

The GC-FID gas flow was set to a 25:1 split flow, with detector flow at approximately 1.75 ml/min, split set at approximately 44 ml/min, and the septum purge set at 3 ml/min. The carrier gas was hydrogen, and the auxiliary gas was nitrogen. All analytical samples were prepared in triplicate and injected in triplicate to monitor for precision in preparation and instrument performance.

### Statistical analysis

The standard error of the mean (SEM) was determined for each set of concentrations or peak areas from each sample. Data are expressed as the mean ± SEM and comparison of means was conducted using a two tailed *t*-test for paired data when differences were observed. The mean values were considered significantly different if *p* < 0.05. Statistical analyses were performed with Microsoft Excel (2022).

## Data Availability

The original contributions presented in the study are included in the article/Supplementary Material, further inquiries can be directed to the corresponding author.
